# Research Progress and Hotspot Evolution Analysis of Landscape Microclimate: Visual Analysis Based on CNKI and WOS

**DOI:** 10.3390/ijerph192215118

**Published:** 2022-11-16

**Authors:** Han Xu, Xinya Lin, Kailong Shi, Shumeng Lin, Guorui Zheng, Qiyue Wang, Jianwen Dong, Minhua Wang

**Affiliations:** 1 College of Landscape Architecture and Art, Fujian Agriculture and Forestry University, 15 Shangxiadian Rd., Fuzhou 350002, China; 2 Engineering Research Center for Forest Park of National Forestry and Grassland Administration, 63 Xiyuangong Rd., Fuzhou 350002, China

**Keywords:** landscape microclimate, CiteSpace, knowledge graph, visualization analysis

## Abstract

With the increasing requirements of healthy habitat environments, landscape microclimates have been widely concerned. To comprehensively grasp the development history and research status of the landscape microclimates in China and other countries, CiteSpace software was used to comparatively analyze and visually present the literature related to landscape microclimates in CNKI and WOS databases for the past 20 years. The results show that: (1) The number of publications on landscape microclimate research shows an increasing trend in China and other countries, and the number of publications increased significantly after 2016. Although the number of publications by Chinese scholars is less than that of foreign scholars, they have developed rapidly in recent years, and their international influence has increased significantly. (2) A positive exchange has been formed among international scholars, and the number of collaborative studies has been increasing. China’s microclimate research has formed relatively stable teams that have conducted numerous studies in the fields of urban communities, street greening, and plant communities, respectively. Although the links between research teams and institutions in China and other countries are relatively close, they still need to be further strengthened. (3) In the past decade, the theoretical system of landscape microclimates has been improved, and the research themes have become more concentrated, but it still has remained close to the early basic research. (4) Future research will remain centered on “mitigating the urban heat island effect” and “optimizing human heat perception”. New topics such as “biodiversity”, “carbon cycle”, and “climate change” have been added. In conclusion, the research needs to continue to explore the evaluation system of microclimates and verify the evaluation index of outdoor thermal comfort for human thermal adaptation in different regions. The standards and systems of human habitat adapted to different regional characteristics should be formed.

## 1. Introduction

At the United Nations General Assembly’s 75th session on 22 September 2020, General Secretary Xi Jinping declared that “carbon dioxide emissions will strive to peak by 2030 and to achieve carbon neutrality by 2060“ [1]. It provides an important strategic guideline for landscape microclimate research [2]. Facing the deterioration of urban climates, landscape architecture can mitigate and adjust to the problems and challenges brought by climate changes through the reasonable arrangement of landscape factors. It improves the urban heat island effect and creates a comfortable and healthy living environment. In recent years, scholars have focused on the evolution of urban green space structure and function under environmental changes [3], the layout and reconstruction of landscape microclimate factors [4], and the evaluation of human comfort perception (including air quality assessments [5], comfort indexes [6], and tourist trip ratio [7]), which have resulted in a fruitful research findings. Relevant studies on landscape microclimate have been summarized by scholars [8]. However, due to the expanding research depth, there are fewer articles that comprehensively compare and analyze the research contents, hot spots, and trends of landscape microclimate among China and other countries. Therefore, to clarify the research contents on microclimates in landscape architecture, this study uses Citespace to analyze the literature data. The China National Knowledge Infrastructure (CNKI) and Web of Science (WOS) databases were used as the research objects, to collect relevant literature in the past 20 years. Combining qualitative description and quantitative analysis, the research contents, themes, cores, and frontiers of domestic and abroad articles are compared and analyzed. It is expected to provide a reference for future research on landscape microclimate.

## 2. Data Sources and Methods

### 2.1. Data Sources

The literature data were obtained from CNKI and WOS databases. The Chinese literature was selected from CNKI databases of academic journals, master’s, and doctoral databases. Among them, academic journals include five core databases: SCI source journals, EI journals, core journal papers, Chinese Social Sciences Citation Index (CSSCI), and Chinese Science Citation Database (CSCD).

CNKI was used as the source of data for Chinese literature. Based on the experimental study site, search articles were TS = (landscape garden OR garden OR residential area OR park OR community OR square OR green space OR campus OR university or street OR plant) AND TS = (microclimate). A total of 964 relevant papers were obtained. After further reading and screening, 760 valid articles were finally collected.

WOS core collection was used as the data source for foreign literature. The search formula was: TS = (microclimate garden) OR TS = (microclimate park) OR TS = (microclimate plaza) OR TS = (microclimate square) OR TS = (microclimate campus) OR TS = (microclimate campus) OR TS = (microclimate street) OR TS = (microclimate residential) OR TS = (microclimate outdoor) OR TS = (microclimate open space) OR TS = (microclimate green space) OR TS = (microclimate green space) space) OR TS = (microclimate neighborhood). The time period was from 2002 to 2022, and the type of literature was research articles and reviews (the search deadline was 1 April 2022). A total of 2238 articles were obtained. Excluding invalid and low relevance literature, 2156 valid articles were collected.

### 2.2. Data Analysis

CiteaSpace is a data visualization software based on JAVA program. It can predict the current status and development trend of research in related scientific and technological fields by analyzing the number of literature, authors, institutions, and keywords [9]. Compared with other visualization software, CiteSpace has the advantages of simple operation, multiple mapping, high information content, automatic recognition mapping, and easy interpretation. It can avoid the subjectivity generated by qualitative analysis effectively [10]. In this study, CiteSpace 5.8R3c was used as a research tool to convert the format of the retrieved literature and import it into the CiteSpace software. The graphs were cropped according to their presentation [11], and different node types were selected to visualize and summarize the research progress and frontier trends of landscape microclimates both domestic and abroad, respectively. It includes the number of publications, author team, co-citation, high-frequency keyword clustering, and keyword emergent analysis.

## 3. Results and Analysis

### 3.1. Post Analysis

**Figure 1:**
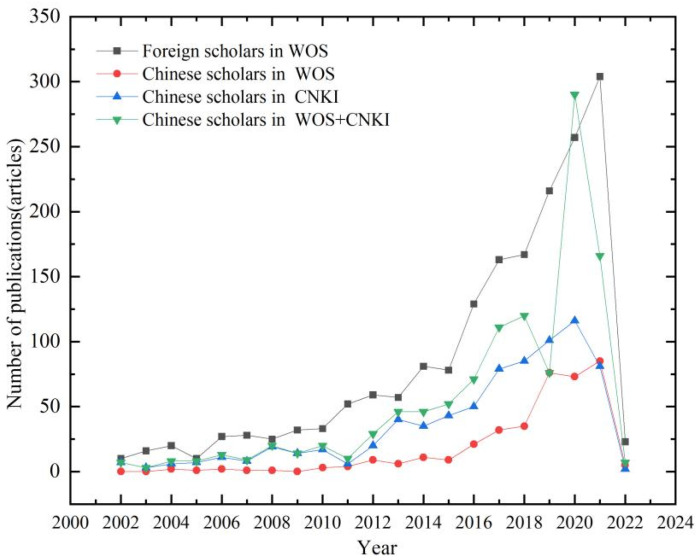
Publication trends.

As shown in Figure 1, the number of landscape microclimate publications in China shows a clear periodical evolution, fluctuating up in 2011 and growing rapidly from 2016 until today. In 2021, the international research of Chinese scholars exceeded those of domestic, and the international contributions of landscape and urban microclimate research increased rapidly, becoming the country with the most achievements in microclimate research. Before 2016, the research in landscape microclimate only carried out a small amount of research around urban greening and building energy consumption. Less attention was paid to the impact of soil water evaporation and vegetation index on microclimates [12]. After 2016, the research showed diversified development. Meanwhile, the Chinese strategy of building an ecological civilization in the 18th National Congress has further promoted the developments of a landscape microclimate in China.

Since 2002, the amount of landscape microclimate publications in other countries have shown a trend of rising–steady–rising again. The 2022 eighth Conference of the Parties (COP8) of the United Nations Framework Convention on Climate Change (UNFCCC) emphasized the severity of climate deterioration and provided an opportunity for the developments of landscape microclimates [13]. With the time development and the increasing global warming, the smooth period is shortening, and the rising period is expanding, which is getting more and more international attention. The development of the international landscape microclimate is in a dividend period and has good prospects for development.

### 3.2. Posting Team Analysis

**Figure 2:**
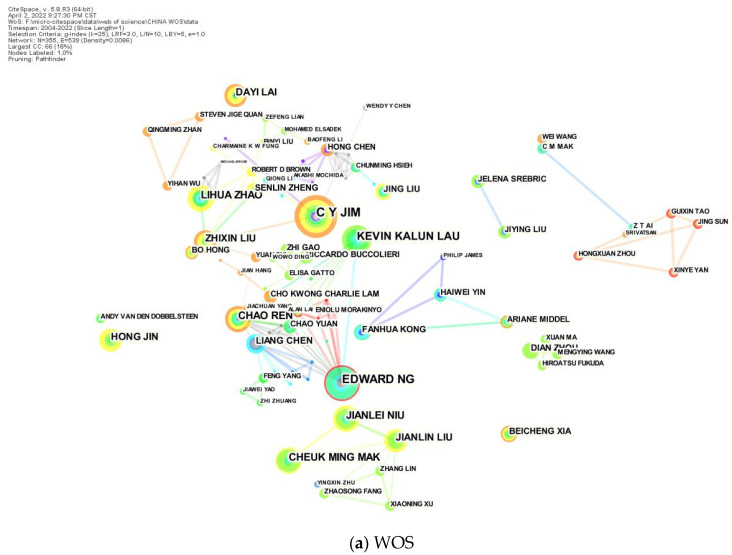

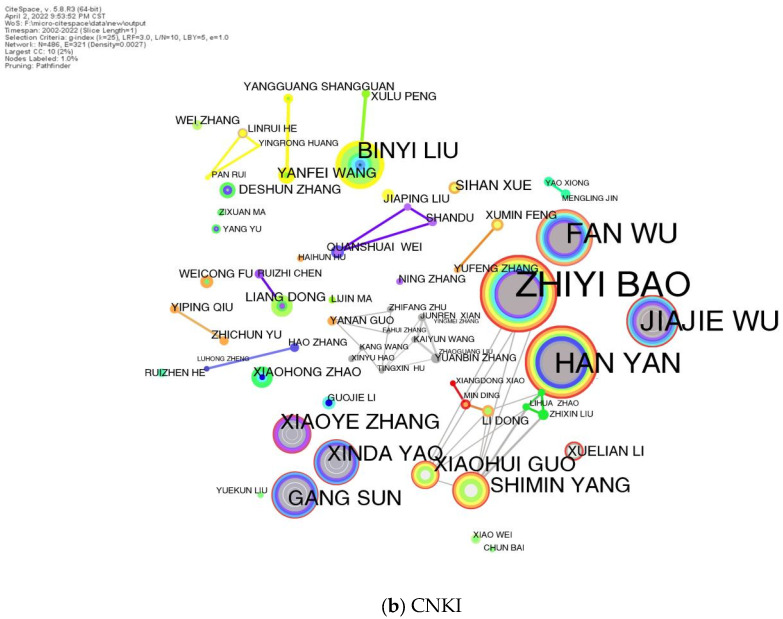
Authorship mapping of Chinese scholars in landscape microclimate.

CiteSpace was used to analyze the data of author teams in landscape and urban microclimate research in China and other countries, which derived the author collaboration graph. The brighter the color and the larger the node size, the more recent the author’s publication date, and the more articles; the darker the color of the line connection, the closer the collaboration between authors. The Chinese graph (CNKI database) has 486 nodes, 321 connections, and a network density of 0.0027 (Figure 2). The Chinese team was mainly composed of Zhiyi Bao–YanhaiFan Wu–Jiajie Wu, Gang Sun–Xingda Yao–Xiaoye Zhang, Liang Dong–Ruiji Chen, and Binyi Liu. Among them, Jim CY’s team had the largest number of publications in English journals in China, and Zhiyi Bao’s team had the largest number of publications in Chinese literature. Zhiyi Bao’s team focused on the spatial and temporal variations of urban green space microclimate and air quality [14], as well as the correlation analysis between thermal comfort and species composition [15]. Liang Dong-Chen Ruiji summarized the role of different landscape elements planning and design on microclimate effects from the perspective of landscape architecture discipline and focused on the practical significance of microclimate on the human living environment from the perspective of human comfort [16]. Liu Bingyi’s team focused on urban streets and environmental users for years, focusing on the relationship between urban street microclimate and users’ travel frequency and overall perception [17,18]; Jim CY’s team used plants as the main research point of cooling and humidification and compared the differences of microclimate effects of different plant communities and species [19]. In conclusion, relatively stable teams have been formed for microclimate environmental restoration research in China, and fruitful research has been carried out in urban communities, street greening, and plant communities.

**Figure 3:**
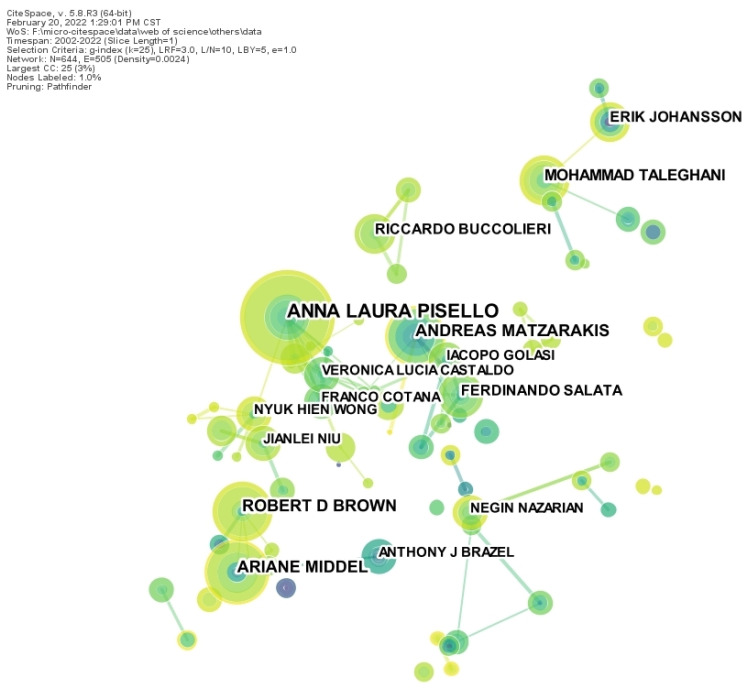
Authorship mapping of foreign scholars in landscape microclimate.

The authorship mapping of other countries (WOS database) had 355 nodes and 539 connections with a network density of 0.0086, which was greater than the network density of the authorship mapping of Chinese publications (Figure 3). International scholars with high publication volumes include Anna LP with 29 occurrences and Ariane M with over 20 occurrences. The articles were published in the period 2015–2017. In terms of the correlation between authors, urban landscape microclimate research was mainly formed by Anna LP’s research team and Robert Db-Ariane M’s research team. Anna LP and Veronica LC, Franco G, and many other scholars conducted research on microclimate in building energy consumption [20]; the Ariane Middel team researched thermal perception. A lot of research work has been carried out, including the heat resistance of people in hot environments [21], the cooling effect of roof gardens [22], and the selection of cold materials [23]. The authorship mapping shows that, in recent years, a positive exchange between international researchers has developed in recent years, with an increasing number of collaborative studies.

### 3.3. Posting Organization Analysis

**Figure 4:**
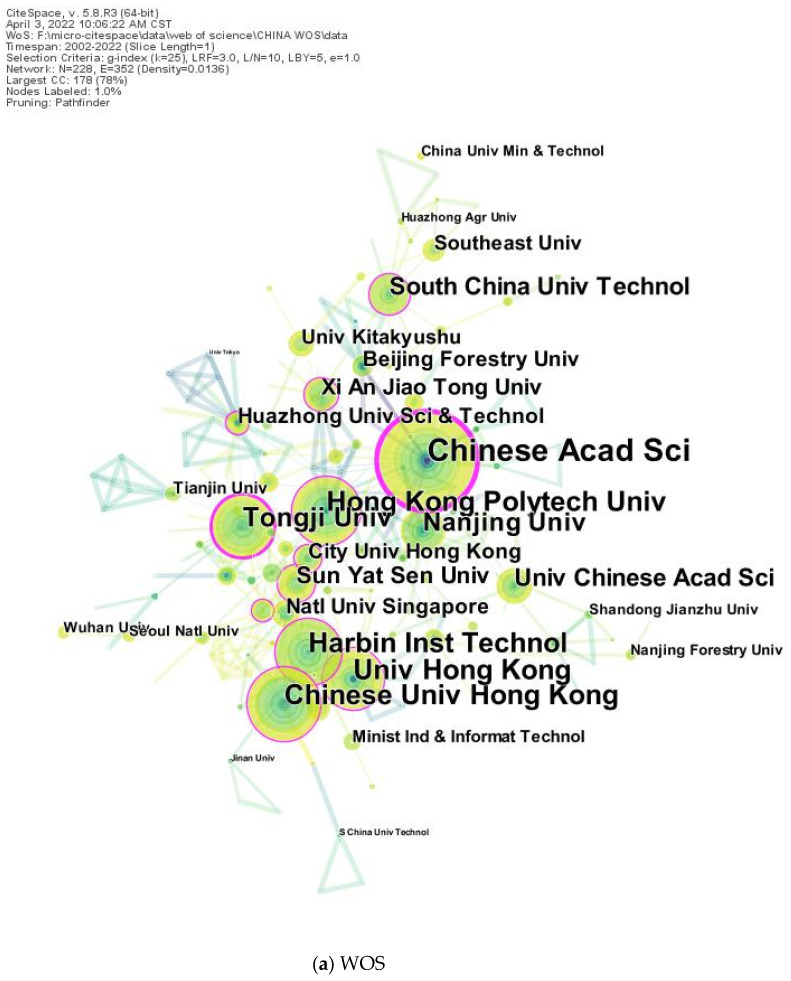

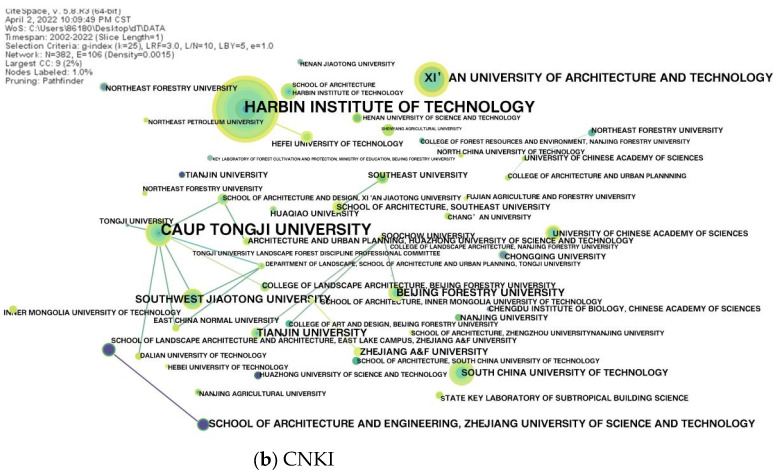
Institutional mapping of landscape microclimate in China.

The visual analysis of the publishing institutions was performed using Citespace. The node size represented the number of publishing institutions, and the nodes with purple outer circles were considered high-centrality nodes, and the centrality was usually greater than 1 [24]. The analysis mapping of Chinese publishing institutions is shown in (Figure 4). In international journals, the leading Chinese institution was the Chinese Academy of Sciences. In domestic journals, the Harbin Institute of Technology had the highest number of publications, with a total of 69 articles. Authors with high publication volumes, such as Leng Hong and Zhao Xiaolong, have become the backbone of research. Universities such as the South China University of Technology, Xi’an University of Architecture and Technology, and Tongji University are also the backbone of landscape microclimate research. In conclusion, the high contributing research institutions were dominated by universities. The publishing institutions mainly focused on the cold regions in the north and the hot and humid regions in the south, forming two research highlands and publishing highly influential research results in their respective research regions.

**Figure 5:**
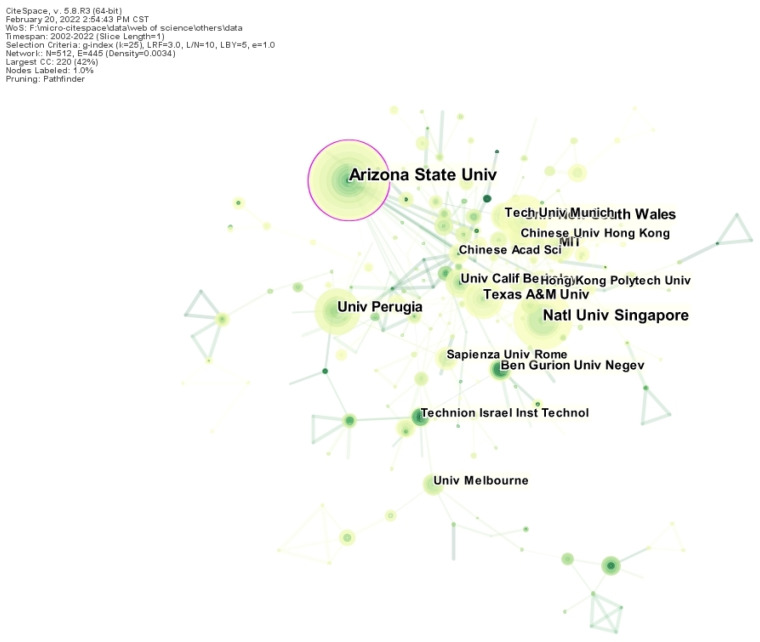
Mapping of foreign publishing institutions in landscape microclimate.

The mapping of foreign publishers had 512 nodes, 445 connections, and a network density of 0.0034 (Figure 5). Arizona State University had the highest number of publications, with more than 30. From the perspective of geography, the countries affiliated with the publishing institutions were mainly located in the United States and Italy. The global distribution was uneven.

### 3.4. Research Hotspots and Trend Analysis

#### 3.4.1. Analysis of Literature Co-Citation

**Figure 6:**
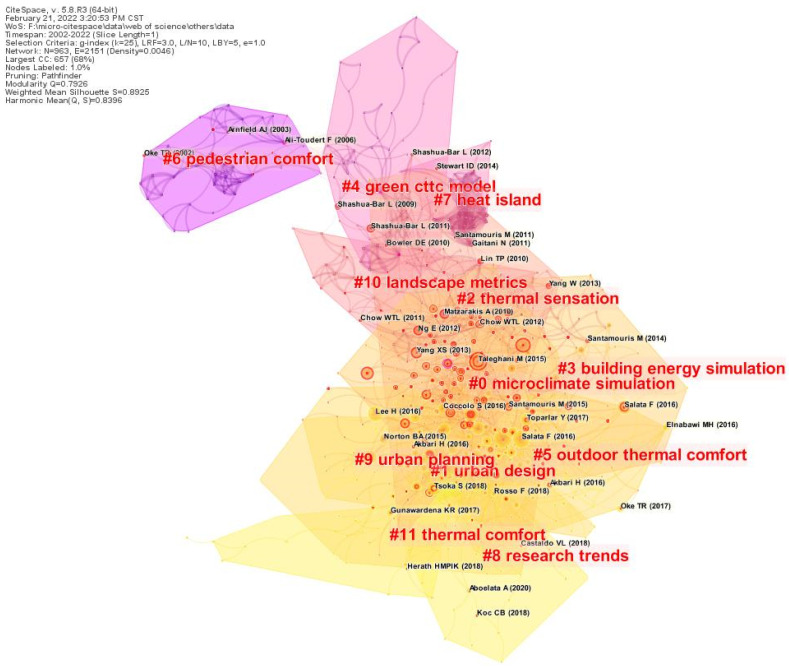
Mapping of co-cited studies in the landscape microclimate research literature.

The literature co-citation analysis provided an overview of the knowledge structure and evolution of research [25]. A literature co-citation mapping of landscape microclimate was constructed based on current literature (Figure 6), and a clustering chart was formed by Citespace software. It provides an overview of the important research results and critical stages in cluster formation during the evolution of landscape microclimate. Since Citespace only supports English literature co-citation analysis currently, this study only analyzed English literature in the WOS core database. The time interval was set from 2002 to 2022. The node type was selected as reference, and the log likelihood ration (LLR) algorithm was used for clustering. In this study, the top 3 keywords were selected as the clustering labels for a detailed description.

##### Microclimate Simulation

Microclimate simulation is a common tool for exploring microclimates at this stage. Compared with traditional microclimate research using measurement instruments experiments, numerical simulation can simulate and predict faster and more accurately. Among the studies with the theme of buildings, Ariane Middel and Yuepeng Wang focused on buildings and studied pollutant dispersion [26] and building energy efficiency [27] in the context of urban heat islands; the research group on the built environment at the Department of Physics, University of Athens, demonstrated that techniques to increase urban albedo and the use of vegetated roofs can present relatively high heat island mitigation potential [28]. In studies with the theme of human comfort, microclimate simulation software has been used to verify design conjectures and analyze the effects of different scenario configurations, including building orientation, material albedo [26], and vegetation layouts [29], on human comfort in outdoor spaces.

##### Urban Design

In urban design, landscape gardening can create a healthy and comfortable urban environment through the rational layout of garden elements. Scholars consider afforestation as one of the effective means to combat urban heat islands and improve thermal comfort. However, plants of different tree species [23] and morphological characteristics [30] have different cooling and humidifying abilities. The cooling and humidifying capacity of tree-dominated community structures are significantly stronger than that of shrubs and ground covers, and its cooling capacity is ranked as follows: tree > tree–shrub–grass > tree–shrub [31]. It has also been found that grasses and water bodies as substrates can increase evapotranspiration and, thus, achieve mitigation of the urban thermal environment [32]. Early urban designs replaced the original natural growing vegetation with materials such as reinforced concrete and asphalt roads. The reflectivity of concrete and asphalt was less than that of green vegetation, and the evaporation effect was poor, causing deterioration of the urban environment. Therefore, the use of soft substrates or water mitigation techniques, such as sprinklers, ponds, and fountains, is recommended to mitigate the microclimate environment [33].

##### Thermal Sensation

Thermal sensation is investigated to minimize the body temperature of pedestrian travel and to improve human comfort. In thermal perception research, the predicted mean vote (PMV) was first proposed by Professor Fanger in Denmark [34]. In order to further deepen the research on human thermal sensation, more relevant human comfort indexes emerged, such as wet bulb black globe temperature (WBGT) [35], physiological equivalent temperature (PET) [36], habitual thermal climate index (UTCI) [37], and standard effective temperature (SET) [38]. It has been found that the perception of the thermal environment is not only related to the physical environment of the microclimate, but also linked to the subjective consciousness of the human body. Once people get the visual enjoyment of an adequately green space environment, thermal sensation decreases with psychological expectation [39]. Therefore, both physical and psychological aspects of thermal sensation are essential concerns in the study of urban microclimate.

#### 3.4.2. Core Keywords and Time Sequence Evolution Characteristics Analysis

Mutability and clustering analysis of keywords for landscape microclimate were performed by Citespace [40]. The bursting and clustering of high-frequency keywords can reflect the research hotspots in a period. Higher bursting values indicate higher levels of attention, reflecting the research hotspots or new research trends of that period of time. Cluster analysis can reveal the association and timeline view among keywords. The clustering words reveal the main components of the research field [41], which can reveal the formation and development of each research hotspot to a certain extent.

The research hotspots of Chinese landscape microclimates are shown in Table 1 and Table 2. The high-frequency keywords published by Chinese scholars in international journals include “outdoor thermal comfort”, “microclimate effect“, “energy use”, “urban design”, “Numerical simulation“, etc. The content of domestic landscape microclimate research is similar to that published in foreign journals, but new research terms, such as “optimal design for cold regions, winter, and traditional villages”, have been added. This indicates that, although the domestic research on landscape microclimate started late, the development speed is not outdated, and the theoretical research system is relatively complete. From the comparison of keyword bursting, Chinese scholars’ keyword bursting published in foreign language journals was almost consistent with that of foreign scholars, but the research in Chinese journals lagged a bit behind and failed to keep pace with the development of international research. From 2018 to date, domestic research on landscape microclimate began to focus on “landscape gardening, thermal comfort, wind environment, cold cities, design strategies“ for research. Changes in microclimate effects are influenced by various factors, such as the surrounding environment, natural geographic conditions, and local historical and humanistic environment, and the thermal environments of cities in different regions need specific analysis. Currently, the research on landscape microclimate in China focuses on the harsh northern region and the hot and humid southern region, and the research objects cover a variety of green space types, such as street green spaces [42], wetland parks [43], ecological campuses [44], leisure plazas [45], outdoor spaces [46], and architectural courtyards [47], and have achieved rich results, in terms of plant landscape elements.

In climate analysis, a microclimate evaluation system adapted to the Chinese characteristics has not been fully formed. Numerical microclimate simulation research in China still needs to rely on foreign research of technology. In the future, further and more comprehensive research on actual measurements and numerical simulations are required. On the premise of meeting the demands of different stakeholders, it is necessary to sort out and summarize the objectives and tasks of landscape gardening climate, explore the evaluation system of microclimates, and verify the evaluation index of outdoor thermal comfort for human thermal adaptation in different regions. Based on the objective human thermal comfort evaluation index, it is necessary to introduce the psychological thermal comfort perception to achieve the unification of physiology and psychology. Additionally, how to balance the relationship between the microclimate environment and visual aesthetic demand in urban green space is also a problem we need to consider.

The clustering of landscape microclimate keywords for other countries are shown in Table 3 and Table 4. In general, “biodiversity”, “vegetation”, “city”, “cfd”, “urbanization”, and “air”, which were formed before 2011, continued to be used until now. “cfd”, “urbanization”, and “air” have been used until now. It indicates that, even though the landscape microclimate research has progressed rapidly, it has not yet departed from the early basic research. Meanwhile, the research has also focused on urban climate adaptable design, neighborhood canyon effect, plant microclimate effect, and numerical simulation. From the keyword clustering analysis, the main basic directions of landscape microclimate research are (1) the impact of the outdoor thermal environment on human comfort, (2) climate simulation of science and technology, and (3) the impact of landscape patterns on landscape microclimate. The dynamic simulation system forms a separate cluster, which mainly focuses on keywords such as energy saving, energy, user demand, and strategy and emphasizes the prediction and simulation of microclimate by science and technology. Therefore, landscape microclimate prediction by numerical simulation will become one of the development trends of the discipline in the future. To gain insight into the research content of landscape climate analysis from 2011 to the present and to clarify the research hotspots of landscape microclimate, the latter decade is divided into two phases.

From 2012 to 2016, mitigation strategies for the urban heat island effect began to be extensively studied. The cooling and humidifying effects of plants and urban layouts on heat island effect mitigation have been demonstrated. With the expansion of research objects, recreational squares [48], university campuses [49], residential areas [26], and street green spaces [50] began to be focused on. The keywords that appear high in this stage are “landscape”, “green roof”, “land use”, “hot dry climate”, and “green roof”. “hot dry climate”, landscape garden, urban land use, roof garden, and extreme weather gradually become research hotspots.

From 2017 to 2022, testing the accuracy, applicability, and validity of human thermal indicators has become a systematic issue. It becomes another important segment of the landscape microclimate. For outdoor thermal comfort in urban spaces, scholars have proposed different thermal comfort indices. Among the 165 developed human thermal indices, only four indices (PET, PMV, UTCI, SET*) have been widely used in outdoor thermal perception studies [32]. At this stage, the keywords with high emergence are “PET”, “outdoors”, “envi-met”, “hot summer”, “outdoor body comfort”, and “summer”, and they became the main research hotspots. Integrated high-frequency keywords and research hotspots show that the research direction of urban microclimates has changed from objective environmental factors to subjective human comfort, from high-density buildings to various urban sites as the theme of research, and from single landscape element measurement to various landscape garden simulation means. In general, the research system of landscape microclimate has been constantly deepened and improved.

## 4. Discussions

A comprehensive bibliometric analysis of the evolution of the landscape microclimate was conducted based on Citespace software. Landscape microclimate has become an important topic for enhancing the habitat and has achieved fruitful achievements.

First, we found that the authors and institutions represented in the landscape microclimate were associated. Most of them were university researchers or professors. Therefore, we can assume that universities may devote more financial, and other, resources to help scholars to pursue their research. It also confirms the finding that the major research institutions for landscape microclimates, both domestic and abroad, are universities. In addition, the research dimension of landscape microclimate is always closely related to geographical location, climate, and population. For example, the harsh northern region and the humid and hot southern region are the most productive regions for landscape microclimate research in China.

Second, the study organized the complete development lineage of landscape microclimates. It was found that landscape microclimate started early and had a rapid development trend, with a relatively complete theoretical system. The high-cited keywords of the literature emphasized the relationship between landscape microclimate and healthy cities, which indicates the future research direction. For example, many scholars have studied how to improve microclimate environments to provide comfortable outdoor open spaces by combining simulation software with measured data in recent studies [51]. Popular software included ENVI-met, CFD, ECOTECT, PHOENICS, Fluent, Rayman, etc. Among them, ENVI-met was the first simulation software specialized for urban microclimate research. It can be seen that software simulation methods have become the main research methods for microclimate studies in recent years [52], which has been confirmed in the articles.

Last, but not least, the research focused on landscape microclimate differed from domestic and abroad. Chinese scholars preferred to start from the research objects, which were divided into the following categories [53]. At the large scale, microclimates were explored from the perspectives of urban morphology and the layout of buildings in high-density urban centers, taking into account the heat island effect, building energy conservation, and pollutant dispersion in cities [54]. At the medium scale, the studies focused on sites of urban parks, residential areas [55], waterfront areas [56], campuses [57], and classical gardens [58]. At the small scale, streets [59], courtyards [60], and plant communities were the main focuses [61]. Compared to China, international scholars concentrated on the relationship between different landscape factors and microclimates from an environmental perspective, such as architectural layout, spatial form, substrate, water bodies, and plant communities [62]. Alternatively, they explored the relationship between using people and microclimates from the human perspective, such as crowd behaviors, perceptual mechanisms, and thermal comfort [63]. Therefore, they are more adept at developing various comfort measures for different areas [64].

## 5. Conclusions

A visual comparative analysis of the landscape microclimate literature published both domestically and abroad in the last 20 years was conducted by Citespace software, and the following conclusions were drawn.

(1) The analysis of the number of publications shows that the number of landscape microclimate research, both domestic and abroad, has shown a sustained increasing trend since 2002, and the number increased significantly after 2016. Although the number of articles published by Chinese scholars on microclimate research was less than that of foreign scholars, it has developed rapidly and increased significantly in international influence over recent years.

(2) The analysis of author co-occurrence shows that international scholars have formed a positive exchange among themselves. The number of collaborative research has increased. Microclimate research in China has also formed relatively stable teams, which have carried out fruitful research in the fields of urban communities, street greening, and plant communities, respectively.

(3) From the keyword clustering analysis, it can be seen that the main basic directions of abroad landscape microclimate research are (1) the influence of outdoor thermal environment on human comfort; (2) numerical simulation of science and technology; (3) the influence of local microclimate among landscape factors. The research hotspots published by Chinese scholars in international journals were consistent with foreign scholars.

(4) The future research trends of landscape microclimate include the following. (1) Mitigation of the urban heat island effect and optimization of human thermal perception will remain to be studied. However, it is no longer limited to analyzing the cooling and humidifying effects of plants, but more to explore the correlation between different landscape factors and human comfort. Alternatively, it can determine the best microclimate effect model for the study sites, based on the healthy city. (2) With the emergence of hotspots for habitat improvement, the conservation and development of biodiversity has also become an important concern. It includes “synergistic relationship between microclimate and biodiversity at regional scales”, “creation of suitable environment for different biological species at local scales”, and “prediction study on the relationship between climate change and biodiversity in cities”. (3) In recent years, how to reduce urban energy consumption, increase urban greenery, and create a comfortable microclimate environment through carbon cycle has become another research hotspot. In the future, the tasks and goals of landscape microclimate need to be sorted out and summarized under the premise of meeting the requirements of different stakeholders. It is necessary to explore the evaluation systems of microclimates and verify the evaluation indexes of outdoor thermal comfort for human thermal adaptation in different regions. Habitat standards and systems adapted to different regional characteristics should be formed as soon as possible.

## Figures and Tables

**Table 1:** Cluster of high-frequency keywords by Chinese scholars.

Cluster (CNKI)	High-Frequency Keywords	Cluster (China-Wos)	High-Frequency Keywords
#0 Microclimate	Microclimate; Plant community; Optimization strategy; Temperature and humidity; Building energy efficiency; Building energy consumption; Energy-efficient design; Plant gardening; Wind speed; Ficus fine-leaved;	#0 CFD	Thermal comfort; Outdoor thermal comfort; Numerical simulation; Cfd; Ventilation; Air; Flow; Cfd simulation; Air flow; Sky view factor;
#1 Microclimate effect	Microclimate effect; Habitat; Residential architecture; Urban forest; Vertical greening; Small habitat; Street space; Guangzhou city; Effect; Pruning;	#1 Outdoor thermal comfort	Design; Simulation; Comfort; Performance; Street canyon; Energy; Mitigation; Urban; Summer; Outdoor;
#2 Landscape garden	Landscape gardening; Spatial form; Thermal comfort; Urban square; Traditional village; Urban street; Urban settlement; Summer; Winter; Settlement;	#2 Urban heat mitigation	Energy use; Balance; Solar radiation; Nonwoven; Street; Scale; Island mitigation strategy; Shade tree; Residential district; Classification;
#3 Ecology	Hilly area; Ecological architecture; Integrated characteristics; Interactive space; Energy saving; Ecology; Environment; Primary and secondary schools; Climatic conditions; Climate;	#3 Microclimate effect	2003 heat wave; Green space; Air quality; Arrangement; Health; Shaw; Temperature; CO^2^ Emission; Heat island; Pattern;
#4 Thermal comfort	Xiamen; Utci; Residential district; Waterscape design; Building layout; Climate design; Nanjing; Revival design; Low-energy building; Environment;	#4 Landscape metrics	Geometry; Forest amenity; Lodz; Graphene ink; Impervious surface; Heat island intensity; Land use; City; Aspect ratio; Valuation;
#5 Human comfort	Optimization study; Physical activity; High-density settlement; Plaza; Micro environment; Comfort; Landscaping; Simulation analysis; Cold land; Experience;	#5 Outdoor thermal comfort	Behavior; Human health; Space; Urban space; Adaptive model; Sensation; Canopy stomatal conductance; American crow; Climate index utci; Heat wave;

**Table 2:** Keywords bursting information table of Chinese landscape microclimate.

**Keywords Burst Information of Chinese Scholars in Foreign Journals**					
**Keywords**	**Year**	**Strength**	**Begin**	**End**	**2002–2022**
rain forest	2002	7.15	2002	2016	▃ ▃ ▃ ▃ ▃ ▃ ▃ ▃ ▃ ▃ ▃ ▃ ▃ ▃ ▃ ▂ ▂ ▂ ▂ ▂ ▂
community	2002	4.97	2003	2016	▂ ▃ ▃ ▃ ▃ ▃ ▃ ▃ ▃ ▃ ▃ ▃ ▃ ▃ ▃ ▂ ▂ ▂ ▂ ▂ ▂
ecosystem	2002	3.91	2003	2014	▂ ▃ ▃ ▃ ▃ ▃ ▃ ▃ ▃ ▃ ▃ ▃ ▃ ▂ ▂ ▂ ▂ ▂ ▂ ▂ ▂
landscape	2002	4.52	2004	2014	▂ ▂ ▃ ▃ ▃ ▃ ▃ ▃ ▃ ▃ ▃ ▃ ▃ ▂ ▂ ▂ ▂ ▂ ▂ ▂ ▂
behavior	2002	3.7	2005	2014	▂ ▂ ▂ ▃ ▃ ▃ ▃ ▃ ▃ ▃ ▃ ▃ ▃ ▂ ▂ ▂ ▂ ▂ ▂ ▂ ▂
water	2002	3.6	2007	2009	▂ ▂ ▂ ▂ ▂ ▃ ▃ ▃ ▂ ▂ ▂ ▂ ▂ ▂ ▂ ▂ ▂ ▂ ▂ ▂ ▂
soil	2002	6.04	2009	2016	▂ ▂ ▂ ▂ ▂ ▂ ▂ ▃ ▃ ▃ ▃ ▃ ▃ ▃ ▃ ▂ ▂ ▂ ▂ ▂ ▂
plant	2002	4.44	2009	2013	▂ ▂ ▂ ▂ ▂ ▂ ▂ ▃ ▃ ▃ ▃ ▃ ▂ ▂ ▂ ▂ ▂ ▂ ▂ ▂ ▂
pattern	2002	3.8	2009	2015	▂ ▂ ▂ ▂ ▂ ▂ ▂ ▃ ▃ ▃ ▃ ▃ ▃ ▃ ▂ ▂ ▂ ▂ ▂ ▂ ▂
growth	2002	6.55	2010	2015	▂ ▂ ▂ ▂ ▂ ▂ ▂ ▂ ▃ ▃ ▃ ▃ ▃ ▃ ▂ ▂ ▂ ▂ ▂ ▂ ▂
temperature	2002	4.71	2010	2011	▂ ▂ ▂ ▂ ▂ ▂ ▂ ▂ ▃ ▃ ▂ ▂ ▂ ▂ ▂ ▂ ▂ ▂ ▂ ▂ ▂
surface	2002	3.63	2011	2017	▂ ▂ ▂ ▂ ▂ ▂ ▂ ▂ ▂ ▃ ▃ ▃ ▃ ▃ ▃ ▃ ▂ ▂ ▂ ▂ ▂
land use	2002	4.25	2012	2016	▂ ▂ ▂ ▂ ▂ ▂ ▂ ▂ ▂ ▂ ▃ ▃ ▃ ▃ ▃ ▂ ▂ ▂ ▂ ▂ ▂
outdoor	2002	4.36	2018	2019	▂ ▂ ▂ ▂ ▂ ▂ ▂ ▂ ▂ ▂ ▂ ▂ ▂ ▂ ▂ ▂ ▃ ▃ ▂ ▂ ▂
energy performance	2002	3.92	2018	2019	▂ ▂ ▂ ▂ ▂ ▂ ▂ ▂ ▂ ▂ ▂ ▂ ▂ ▂ ▂ ▂ ▃ ▃ ▂ ▂ ▂
summer	2002	3.97	2019	2020	▂ ▂ ▂ ▂ ▂ ▂ ▂ ▂ ▂ ▂ ▂ ▂ ▂ ▂ ▂ ▂ ▂ ▃ ▃ ▂ ▂
envi-met	2002	4.44	2020	2022	▂ ▂ ▂ ▂ ▂ ▂ ▂ ▂ ▂ ▂ ▂ ▂ ▂ ▂ ▂ ▂ ▂ ▂ ▃ ▃ ▃
**Keyword Burst Information of Chinese Scholars in Domestic Journals**					
**Keywords**	**Year**	**Strength**	**Begin**	**End**	**2002** **–2022**
microclimate	2002	7.83	2006	2009	▂ ▂ ▂ ▂ ▃ ▃ ▃ ▃ ▂ ▂ ▂ ▂ ▂ ▂ ▂ ▂ ▂ ▂ ▂ ▂ ▂
landscape gardening	2002	4.72	2018	2019	▂ ▂ ▂ ▂ ▂ ▂ ▂ ▂ ▂ ▂ ▂ ▂ ▂ ▂ ▂ ▂ ▃ ▃ ▂ ▂ ▂
wind environment	2002	3.34	2018	2019	▂ ▂ ▂ ▂ ▂ ▂ ▂ ▂ ▂ ▂ ▂ ▂ ▂ ▂ ▂ ▂ ▃ ▃ ▂ ▂ ▂
thermal comfort	2002	4.02	2019	2020	▂ ▂ ▂ ▂ ▂ ▂ ▂ ▂ ▂ ▂ ▂ ▂ ▂ ▂ ▂ ▂ ▂ ▃ ▃ ▂ ▂
cold land cities	2002	3.65	2019	2022	▂ ▂ ▂ ▂ ▂ ▂ ▂ ▂ ▂ ▂ ▂ ▂ ▂ ▂ ▂ ▂ ▂ ▃ ▃ ▃ ▃
design strategies	2002	3.3	2019	2020	▂ ▂ ▂ ▂ ▂ ▂ ▂ ▂ ▂ ▂ ▂ ▂ ▂ ▂ ▂ ▂ ▂ ▃ ▃ ▂ ▂

**Table 3:** Clustering and temporal evolution of high-frequency keywords in other countries.

Cluster	Year		
	2002–2011	2012–2016	2017–2022
#0 Outdoor thermal comfort	biodiversity (10); vegetation (26); forest (6); community (7); national park (4); pattern (9);	biodiversity (22); vegetation (65); forest (7); community (12); national park (15); pattern (14);	biodiversity (36); vegetation (134); forest (14); community (6); national park (11); pattern (25);
		vulnerability (2); shade (2); management (5);	vulnerability (7); shade (10); management (20);
			ecosystem service (16); greenery (5);
#1 Envi-met	city (13); canyon (5); island (3); air temperature (44); area (13); design (15);	city (48); canyon (9); island (13); air temperature (86); area (28); design (40);	city (135); canyon (34); island (33); air temperature (284); area (50); design (146);
		urban heat island (26); surface temperature (2); hot dry climate (11); mean radiant temperature (10); pet (3);	urban heat island (111); surface temperature (15); hot dry climate (11); mean radiant temperature (52); pet (19);
			utci (17); envi-met (24); morphology (15); aspect ratio (17);
#2 Cfd	cfd (7); air flow (8); air pollution (4); wind (3); street canyon (2); numerical simulation (2);	cfd (13); air flow (21); air pollution (7); wind (4); street canyon (17); numerical simulation (6);	cfd (40); air flow (46) air pollution (21); wind (22); street canyon (83); numerical simulation (23);
		prediction (10); pollution dispersion (2); cfd simulation (4);	prediction (13); pollution dispersion (24); cfd simulation (14);
			mitigation strategy (24); ventilation (45); green infrastructure (23); hot summer (18);
#3 Landscape metrics	flux (5); street (3); park (2); adaptation (1); space (4); health (2);	flux (3); street (9); park (8); adaptation (15); space (24); health (4)	flux (19); street (27); park (46); adaptation (62); space (89); health (37)
		index (23); land surface temperature (5); summer (13);	index (49); land surface temperature (23); summer (43);
			green space (28); sensation (28); physical activity (10);
#4 Trnsys	air (5); building (10); simulation (10); performance (4);	air (8); building (23); simulation (24); performance (28);	air (35); building (62); simulation (126); performance (123);
		demand (2); energy consumption (4); strategy (5);	demand (20); energy consumption (28); strategy (50);
			heat strategy (22); neighborhood (12); microclimate model (8);

**Table 4:** Keyword bursting information table for foreign landscape microclimate research.

Keywords	Year	Strength	Begin	End	2002–2022
rain forest	2002	7.77	2002	2016	▃ ▃ ▃ ▃ ▃ ▃ ▃ ▃ ▃ ▃ ▃ ▃ ▃ ▃ ▃ ▂ ▂ ▂ ▂ ▂ ▂
community	2002	5.28	2003	2016	▂ ▃ ▃ ▃ ▃ ▃ ▃ ▃ ▃ ▃ ▃ ▃ ▃ ▃ ▃ ▂ ▂ ▂ ▂ ▂ ▂
model	2002	3.88	2003	2008	▂ ▃ ▃ ▃ ▃ ▃ ▃ ▂ ▂ ▂ ▂ ▂ ▂ ▂ ▂ ▂ ▂ ▂ ▂ ▂ ▂
ecosystem	2002	3.81	2003	2016	▂ ▃ ▃ ▃ ▃ ▃ ▃ ▃ ▃ ▃ ▃ ▃ ▃ ▃ ▃ ▂ ▂ ▂ ▂ ▂ ▂
area	2002	6.09	2004	2014	▂ ▂ ▃ ▃ ▃ ▃ ▃ ▃ ▃ ▃ ▃ ▃ ▃ ▂ ▂ ▂ ▂ ▂ ▂ ▂ ▂
behavior	2002	3.75	2005	2014	▂ ▂ ▂ ▃ ▃ ▃ ▃ ▃ ▃ ▃ ▃ ▃ ▃ ▂ ▂ ▂ ▂ ▂ ▂ ▂ ▂
radiation	2002	4.42	2007	2016	▂ ▂ ▂ ▂ ▂ ▃ ▃ ▃ ▃ ▃ ▃ ▃ ▃ ▃ ▃ ▂ ▂ ▂ ▂ ▂ ▂
temperature	2002	5.24	2008	2011	▂ ▂ ▂ ▂ ▂ ▂ ▃ ▃ ▃ ▃ ▂ ▂ ▂ ▂ ▂ ▂ ▂ ▂ ▂ ▂ ▂
vegetation	2002	4.89	2008	2012	▂ ▂ ▂ ▂ ▂ ▂ ▃ ▃ ▃ ▃ ▃ ▂ ▂ ▂ ▂ ▂ ▂ ▂ ▂ ▂ ▂
plant	2002	6.37	2009	2013	▂ ▂ ▂ ▂ ▂ ▂ ▂ ▃ ▃ ▃ ▃ ▃ ▂ ▂ ▂ ▂ ▂ ▂ ▂ ▂ ▂
soil	2002	6.14	2009	2016	▂ ▂ ▂ ▂ ▂ ▂ ▂ ▃ ▃ ▃ ▃ ▃ ▃ ▃ ▃ ▂ ▂ ▂ ▂ ▂ ▂
pattern	2002	4.2	2009	2015	▂ ▂ ▂ ▂ ▂ ▂ ▂ ▃ ▃ ▃ ▃ ▃ ▃ ▃ ▂ ▂ ▂ ▂ ▂ ▂ ▂
growth	2002	7.36	2010	2015	▂ ▂ ▂ ▂ ▂ ▂ ▂ ▂ ▃ ▃ ▃ ▃ ▃ ▃ ▂ ▂ ▂ ▂ ▂ ▂ ▂
national park	2002	7.25	2011	2015	▂ ▂ ▂ ▂ ▂ ▂ ▂ ▂ ▂ ▃ ▃ ▃ ▃ ▃ ▂ ▂ ▂ ▂ ▂ ▂ ▂
land use	2002	4.53	2012	2016	▂ ▂ ▂ ▂ ▂ ▂ ▂ ▂ ▂ ▂ ▃ ▃ ▃ ▃ ▃ ▂ ▂ ▂ ▂ ▂ ▂
landscape	2002	4.16	2012	2014	▂ ▂ ▂ ▂ ▂ ▂ ▂ ▂ ▂ ▂ ▃ ▃ ▃ ▂ ▂ ▂ ▂ ▂ ▂ ▂ ▂
hot dry climate	2002	3.67	2014	2016	▂ ▂ ▂ ▂ ▂ ▂ ▂ ▂ ▂ ▂ ▂ ▂ ▃ ▃ ▃ ▂ ▂ ▂ ▂ ▂ ▂
convective heat transfer	2002	4.22	2015	2017	▂ ▂ ▂ ▂ ▂ ▂ ▂ ▂ ▂ ▂ ▂ ▂ ▂ ▃ ▃ ▃ ▂ ▂ ▂ ▂ ▂
green roof	2002	4.06	2015	2017	▂ ▂ ▂ ▂ ▂ ▂ ▂ ▂ ▂ ▂ ▂ ▂ ▂ ▃ ▃ ▃ ▂ ▂ ▂ ▂ ▂
Hong Kong	2002	4.19	2016	2018	▂ ▂ ▂ ▂ ▂ ▂ ▂ ▂ ▂ ▂ ▂ ▂ ▂ ▂ ▃ ▃ ▃ ▂ ▂ ▂ ▂
physiological equivalent temperature	2002	3.97	2016	2018	▂ ▂ ▂ ▂ ▂ ▂ ▂ ▂ ▂ ▂ ▂ ▂ ▂ ▂ ▃ ▃ ▃ ▂ ▂ ▂ ▂
outdoor	2002	4.9	2018	2019	▂ ▂ ▂ ▂ ▂ ▂ ▂ ▂ ▂ ▂ ▂ ▂ ▂ ▂ ▂ ▂ ▃ ▃ ▂ ▂ ▂
scale	2002	3.94	2018	2020	▂ ▂ ▂ ▂ ▂ ▂ ▂ ▂ ▂ ▂ ▂ ▂ ▂ ▂ ▂ ▂ ▃ ▃ ▃ ▂ ▂
envi-met	2002	5.23	2020	2022	▂ ▂ ▂ ▂ ▂ ▂ ▂ ▂ ▂ ▂ ▂ ▂ ▂ ▂ ▂ ▂ ▂ ▂ ▃ ▃ ▃
hot summer	2002	4.12	2020	2022	▂ ▂ ▂ ▂ ▂ ▂ ▂ ▂ ▂ ▂ ▂ ▂ ▂ ▂ ▂ ▂ ▂ ▂ ▃ ▃ ▃

## Data Availability

All images in the text were drawn by the author. The data used to support the findings of this study are available from the corresponding author upon request.
